# Trends of the Dementia Burden in South Asia: An Analysis of 2021 Global Burden of Disease Study

**DOI:** 10.1002/agm2.70002

**Published:** 2025-02-26

**Authors:** Shubham Chauhan, Diptismita Jena, Shilpa Gaidhane, Navneet Dev, Ganesh Bushi, G. Padma Priya, Pawan Sharma, Mahakshit Bhat, Shilpa Sharma, M. Ravi Kumar, Aashna Sinha, Quazi Syed Zahiruddin, Muhammed Shabil, Sanjit Sah, Rukshar Syed, Kamal Kundra, Alisha Dash, Hashem Abu Serhan

**Affiliations:** ^1^ Global Center for Evidence Synthesis Chandigarh India; ^2^ Saveetha Medical College and Hospital, Saveetha Institute of Medical and Technical Sciences Saveetha University Chennai India; ^3^ One Health Centre (COHERD), Jawaharlal Nehru Medical College Datta Meghe Institute of Higher Education Wardha India; ^4^ Department of Dermatology Graphic Era Deemed to Be University Dehradun India; ^5^ School of Pharmaceutical Sciences Lovely Professional University Phagwara India; ^6^ Department of Chemistry and Biochemistry, School of Sciences JAIN (Deemed to Be University) Bangalore Karnataka India; ^7^ Department of Sciences Vivekananda Global University Jaipur India; ^8^ Department of Medicine, National Institute of Medical Sciences NIMS University Rajasthan Jaipur India; ^9^ Chandigarh Pharmacy College Chandigarh Group of Colleges‐Jhanjeri Mohali Punjab India; ^10^ Department of Chemistry Raghu Engineering College Visakhapatnam India; ^11^ Division of Research and Innovation, Uttaranchal Institute of Pharmaceutical Sciences Uttaranchal University Vikasnagar India; ^12^ Division of Evidence Synthesis, South Asia Infant Feeding Research Network (SAIFRN), Global Consortium of Public Health and Research Datta Meghe Institute of Higher Education Wardha India; ^13^ University Center for Research and Development, Chandigarh University Mohali Punjab India; ^14^ Medical Laboratories Techniques Department AL‐Mustaqbal University Babil Iraq; ^15^ Department of Paediatrics Dr. D. Y. Patil Medical College, Hospital and Research Centre Pune Maharashtra India; ^16^ Department of Public Health Dentistry Dr. D.Y. Patil Dental College and Hospital, Dr. D.Y. Patil Vidyapeeth Pune Maharashtra India; ^17^ IES Institute of Pharmacy IES University Bhopal Madhya Pradesh India; ^18^ New Delhi Institute of Management Delhi India; ^19^ KIIT School of Biotechnology KIIT University Bhubaneswar India; ^20^ Department of Ophthalmology Hamad Medical Corporation Doha Qatar

**Keywords:** Alzheimer's disease, dementia, epidemiology, GBD 2021, global health, public health policy, South Asia

## Abstract

**Objectives:**

This study aims to analyze the trends in the burden of Alzheimer's Disease and Other Dementias (ADoD) in South Asia from 1990 to 2021, focusing on incidence, prevalence, mortality, and Disability‐Adjusted Life Years (DALYs). The objective is to identify key risk factors, such as metabolic and behavioral health risks, and assess regional variations in the burden of ADoD across five South Asian countries India, Pakistan, Bangladesh, Nepal, and Bhutan.

**Methods:**

Data from the Global Burden of Disease (GBD) 2021 report were analyzed using descriptive statistics and join point regression analysis. The analysis evaluated trends in ADoD incidence, prevalence, mortality, and DALYs across five South Asian countries, focusing on health risk factors, including high body mass index, behavioral and metabolic risks.

**Results:**

A slight decrease in incidence rates from 80.57 to 79 per 100,000 was observed, alongside a significant increase in mortality rates from 14.11 to 17.2 per 100,000. While prevalence rates experienced a minor decline, Disability‐Adjusted Life Years (DALYs) rose from 272.02 to 308.27 per 100,000, reflecting an increasing burden of the disease. Notably, Nepal significantly reduced its incidence rates, while Pakistan saw an increase in mortality rates. In South Asia, the highest‐ranking risk factor is metabolic risks, followed by high fasting plasma glucose (FPG).

**Conclusions:**

The growing burden of ADoD in South Asia necessitates targeted public health strategies addressing key risk factors, with metabolic health risks being a primary contributor. Public health interventions should focus on the most affected populations, particularly the elderly and females, to mitigate the increasing impact of dementia across the region.

## Graphical abstract

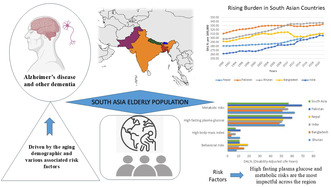

## 1 Introduction

Dementia is a syndrome primarily characterized by a decline in cognitive functions, with Alzheimer's disease (AD) being the most common type [1]. Dementia predominantly affects older adults, with incidence and prevalence rates increasing significantly with age, a trend particularly pronounced in low and middle income countries (LMIC). This has escalated into a serious global public health concern, imposing substantial economic and disease burdens on societies and families [2, 3]. Dementia represents an escalating global health challenge, particularly in regions experiencing rapid population aging. Given the substantial and growing global impact of dementia, particularly in South Asia, this study aims to analyze the epidemiological patterns of dementia in South Asia, focusing on changes in disease burden over time and the influence of major health risk factors. According to the Global Burden of Disease (GBD) study, approximately 57.4 million people globally were living with dementia in 2019, and this number is projected to increase dramatically, reaching 152.8 million by 2050 [4]. Clinically, AD manifests through a range of symptoms including memory disorders, cognitive impairments, executive dysfunction, and changes in personality and behavior. Additionally, many patients experience co‐occurring psychiatric symptoms [5, 6]. While detailed care and medication can temporarily alleviate some of these symptoms, there are currently no definitive measures to prevent or cure AD [7]. South Asia, a region characterized by rapid demographic transitions and economic growth, is particularly vulnerable to this rising burden. The region is home to approximately one‐quarter of the world's population, and the projected increase in dementia cases is expected to have profound implications for public health systems, which are often under resourced and ill‐equipped to manage the growing demand for dementia care in 2019 [8]. Moreover, the cultural context in South Asia, where cognitive decline is often seen as a normal part of aging, further complicates efforts to diagnose and treat dementia early, leading to delayed interventions and poorer outcomes [9]. Despite the significant advances in the diagnosis and treatment of dementia in high‐income countries, South Asian nations struggle with inadequate healthcare infrastructure, limited public awareness, and significant socio‐economic barriers that prevent effective management of dementia [9, 10, 11]. These challenges are exacerbated by the rapid aging of the population, increasing life expectancy, and a rising prevalence of risk factors such as cardiovascular diseases, diabetes, and hypertension [8]. The lack of targeted public health policies and the absence of a coherent strategy to address dementia in the region underscores the critical need for comprehensive research that provides a detailed understanding of the current and future burden of dementia in South Asia.

The anticipated rise in dementia cases in South Asia will have significant implications for global public health, particularly in terms of resource allocation, healthcare planning, and policy development [12, 13]. The economic impact of dementia is substantial, with care costs expected to soar as more individuals require long‐term care. Moreover, dementia imposes significant strains on care systems and families, encompassing direct medical costs, direct social costs, and indirect costs related to informal caregiving and lost productivity [11, 14, 15]. Understanding the trends of dementia in South Asia is crucial for informing both regional and global strategies aimed at mitigating the impact of this growing health challenge. This study aims to analyzing the changes in incidence, prevalence, mortality and DALY of ADoD across five South Asian countries spanning from 1990 to 2021. It explores the role of various health risk factors, such as high body mass index, smoking, and metabolic risks, in contributing to regional disparities in the epidemiology of dementia. The focus of this investigation is on assessing the temporal and geographical variations in dementia metrics.

## 2 Methods

### 2.1 Data Sources and Variables

Data were extracted from the Global Health Data Exchange (GHDx) platform (https://vizhub.healthdata.org/gbd‐results/) which provides comprehensive, standardized estimates of incidence, prevalence, mortality, and DALYs for various diseases and conditions across 195 countries, including South Asian nations such as India, Pakistan, Nepal, Bangladesh, and Bhutan [16, 17]. The methodology adopted for this study involves a systematic approach that integrates data from multiple sources, including vital registration systems, published scientific literature, and health surveys, to provide reliable estimates for dementia prevalence and its associated burden [8]. It was assumed that dementia onset occurs after age 40 due to its chronic and progressive nature, coupled with its marked rarity among individuals below this age threshold [18, 19].

### 2.2 Statistical Analysis

The statistical analysis for this study was conducted using a combination of descriptive statistics and join point regression analysis to evaluate the temporal trends in ADoD metrics across South Asia from 1990 to 2021. In descriptive analysis, data spanning from 1990 to 2021 were utilized to evaluate trends in ADoD across South Asia. Metrics including incidence, prevalence, mortality rates, years lived with disability (YLD), years of life lost (YLL), and DALYs were examined. Descriptive statistics, specifically means and standard deviations, were computed for each metric to summarize the data over the 32‐year study period for an analytical review of the temporal trends in ADoD metrics within the region.

Join point regression was specifically utilized to identify significant changes in the trends of age‐standardized incidence rates (ASIR), prevalence, mortality, and DALYs. This method is highly effective in detecting points where the rate of change shifts significantly. The join point analysis was conducted using Joinpoint software version 5.2.0.0, developed by the National Cancer Institute. The join point model employed a logarithmic approach, which is well‐suited for modeling data that exhibit exponential growth or decay, such as the increasing trends in dementia incidence due to aging populations. The number of join points was determined using a permutation test that identified the optimal model by minimizing the Bayesian Information Criterion (BIC). This approach allowed for the detection of multiple significant changes in trends over the 31‐year study period. Statistical significance was assessed using a *p* value threshold of 0.050, and 95% confidence intervals were calculated for the annual percent change (APC) to ensure the robustness of the identified trends.

## 3 Results

Table 1 shows the trends in ADoD metrics in South Asia from 1990 to 2021. The incidence rate decreased slightly from 80.57 to 79 per 100,000, while prevalence rates declined from 446.47 to 437.07 per 100,000 (Table 1). Mortality rates increased significantly, rising from 14.11 to 17.2 per 100,000. YLD dropped marginally from 87.61 to 86.55 per 100,000, while YLL rose from 184.41 to 221.73 per 100,000. Overall, DALYs increased from 272.02 to 308.27 per 100,000. The mean values for these metrics remained relatively stable with minor fluctuations over the study period.

**TABLE 1:** Descriptive analysis of temporal variations in ADoD metrics across South Asia from 1990 to 2021.

Year	Incidence rate	Prevalence rate	Mortality rate	YLD	YLL	DALY
1990	80.57 (69.5–92.11)	446.47 (386.42–510.25)	14.11 (3.23–40.84)	87.61 (114.19–60.12)	184.41 (524–42.34)	272.02 (126.23–615.5)
1991	80.37 (69.36–91.89)	445.56 (385.54–509.5)	14.15 (3.22–40.16)	87.43 (114.02–60.08)	184.99 (527.74–42.1)	272.42 (125.73–618.92)
1992	80.17 (69.22–91.67)	444.57 (384.82–508.62)	14.3 (3.28–40.53)	87.23 (113.94–59.89)	186.69 (521.98–42.91)	273.91 (126.46–616.05)
1993	79.96 (69.06–91.45)	443.54 (384.28–507.35)	14.38 (3.29–40.68)	87.06 (113.53–59.61)	187.68 (527.59–42.96)	274.74 (126.6–618.56)
1994	79.76 (68.91–91.24)	442.55 (383.59–506.31)	14.48 (3.29–41.44)	86.85 (113.26–59.52)	188.88 (532.26–42.43)	275.73 (125.42–623.17)
1995	79.56 (68.78–91.09)	441.54 (382.74–505.61)	14.72 (3.36–42.29)	86.65 (113.04–59.37)	191.65 (536.93–43.48)	278.3 (126.14–630.62)
1996	79.32 (68.57–90.75)	440.28 (382.05–504.16)	14.74 (3.34–42.29)	86.44 (112.9–59.31)	192 (539.47–43.29)	278.44 (126.77–629.96)
1997	79.05 (68.33–90.37)	438.81 (381.11–502.05)	14.72 (3.39–41.99)	86.22 (112.54–59.2)	191.84 (538.37–44.19)	278.06 (126.18–628.53)
1998	78.78 (68.09–90.03)	437.32 (379.83–500.27)	14.79 (3.39–42.1)	85.98 (112–58.76)	192.83 (542.31–43.85)	278.8 (126.75–632.58)
1999	78.52 (67.86–89.76)	435.92 (378.44–498.16)	14.72 (3.34–41.12)	85.78 (112–58.75)	192.2 (535.8–43.71)	277.97 (126.08–625.71)
2000	78.33 (67.68–89.58)	434.87 (377.39–496.55)	14.72 (3.45–41.49)	85.6 (111.69–58.59)	192.27 (534.46–44.09)	277.87 (125.06–623.82)
2001	78.15 (67.51–89.39)	433.98 (376.79–495.44)	14.88 (3.42–41.94)	85.45 (111.25–58.61)	194.07 (529.73–44.06)	279.52 (125.06–619.21)
2002	77.94 (67.32–89.14)	432.89 (376–494.1)	15.02 (3.45–42.19)	85.26 (111.34–58.45)	195.71 (543.2–44.28)	280.97 (126.07–637.3)
2003	77.73 (67.12–88.88)	431.76 (375.15–492.77)	15.23 (3.61–43.07)	85.08 (110.86–58.58)	197.87 (550.25–45.58)	282.95 (125.98–644.08)
2004	77.55 (66.96–88.66)	430.82 (374.14–491.68)	15.38 (3.54–43.36)	84.92 (110.92–58.32)	199.37 (542.29–45.02)	284.28 (127.45–635.84)
2005	77.43 (66.85–88.52)	430.18 (373.34–490.8)	15.45 (3.54–43.49)	84.82 (110.86–58.28)	200.26 (550.04–44.7)	285.08 (127.35–646.82)
2006	77.39 (66.86–88.47)	429.85 (373.2–490.68)	15.62 (3.65–43.93)	84.77 (110.96–58.26)	202.01 (556.63–45.91)	286.77 (128.34–652.18)
2007	77.4 (66.9–88.5)	429.7 (373.2–490.41)	15.83 (3.69–44.25)	84.74 (110.83–58.24)	204.39 (561.34–46.57)	289.13 (129.7–656.23)
2008	77.44 (66.96–88.55)	429.61 (372.95–490.3)	15.89 (3.67–45.05)	84.69 (110.76–58.19)	205.2 (567.69–47.5)	289.88 (128.78–648.35)
2009	77.46 (67–88.59)	429.46 (372.54–490.29)	15.8 (3.7–44.39)	84.67 (110.79–58.23)	204.42 (565.07–47.36)	289.1 (129.55–643.1)
2010	77.43 (67–88.58)	429.13 (372.53–490.1)	15.85 (3.71–44.21)	84.63 (110.37–57.94)	205.11 (557.56–46.34)	289.74 (129.92–642.34)
2011	77.21 (66.78–88.28)	427.86 (371.16–488.79)	15.9 (3.78–43.69)	84.43 (110.33–58.06)	205.79 (548.48–47.21)	290.22 (128.9–643.64)
2012	76.76 (66.37–87.71)	425.43 (368.64–486.11)	15.98 (3.79–43.39)	84.04 (109.92–57.7)	206.53 (550.86–47.32)	290.58 (129.02–649.33)
2013	76.23 (65.89–87.06)	422.64 (365.8–483)	16.55 (3.85–45.98)	83.58 (109.33–57.16)	212.15 (575.39–48.98)	295.73 (129.63–661.77)
2014	75.81 (65.5–86.54)	420.38 (363.61–480.41)	16.86 (3.93–46.5)	83.23 (108.72–56.9)	215.2 (579.2–50.49)	298.43 (129.94–664.9)
2015	75.61 (65.3–86.29)	419.27 (362.59–479.01)	16.9 (4.04–47.12)	83.1 (108.63–56.92)	216.18 (586.21–51.57)	299.28 (131.36–665.1)
2016	75.55 (65.27–86.23)	418.77 (362.57–478.98)	16.92 (3.98–46.98)	83.08 (108.74–56.83)	216.9 (584.67–51)	299.98 (130.57–676.49)
2017	75.48 (65.23–86.15)	418.15 (362.06–478.55)	17.12 (4.06–48.34)	83.03 (108.61–56.76)	219.72 (601.99–51.93)	302.75 (131.86–687.14)
2018	75.45 (65.22–86.13)	417.7 (361.69–478.47)	17.19 (4.14–47.88)	83.02 (108.77–56.59)	221.01 (605.21–52.42)	304.03 (132.37–685.27)
2019	75.49 (65.26–86.19)	417.71 (361.39–478.93)	17.3 (4.16–48.57)	83.05 (108.94–56.77)	222.32 (609.81–53.64)	305.37 (133.28–693.16)
2020	77.43 (67.07–88.49)	431.48 (373.47–494.19)	17.39 (4.16–48.15)	85.56 (112.23–58.65)	223.42 (616.99–53.51)	308.98 (136.93–705.42)
2021	79 (68.26–90.52)	437.07 (377–500.95)	17.2 (4.1–47.28)	86.55 (113.8–59.27)	221.73 (605.28–52.73)	308.27 (135.52–684.96)
Average (x)	77.82	431.73	15.63	85.14	202.34	287.48
S.D. (σ)	1.54	8.68	1.05	1.41	12.21	11.14
3σ CL (*x* ± 3σ)	(73.20–82.45)	(405.68–457.78)	(12.48–18.78)	(80.91–89.7)	(165.69–238.98)	(254.07–320.89)
Percentage change (1990–2021)	−1.95	−2.11	21.90	−1.21	20.24	13.33

**FIGURE 1:**
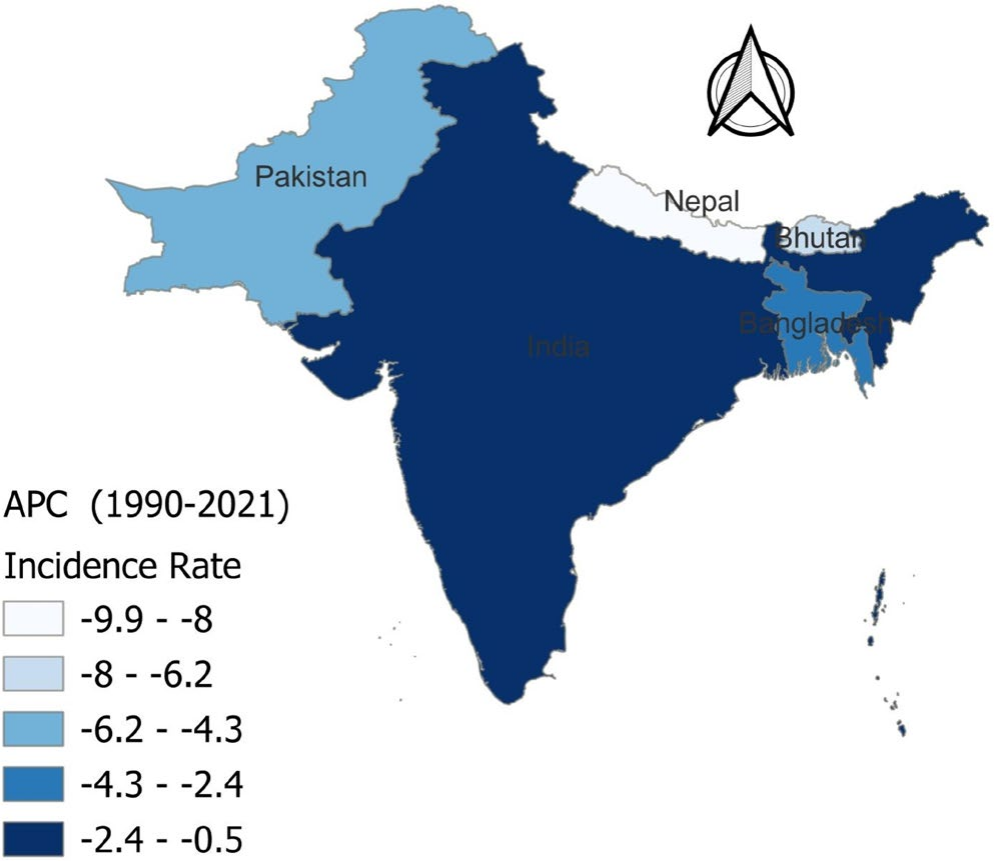
Annual percentage change (APC) in incidence rate of Alzheimer's disease and other dementias in South Asia (1990–2021).

The APC in the incidence rates of ADoD from 1990 to 2021 reveals notable differences among the five South Asian countries (Figure 1). Nepal experienced the most substantial decline, with a percentage change of −9.91%, leading the region in reducing dementia incidence. Pakistan and Bhutan followed with declines of −5.64% and −6.43%, respectively, showing moderate reductions. Bangladesh recorded a smaller decrease of −3.07%, while India exhibited the least change with a marginal decrease of only −0.53%.

The analysis of AAPC in ADoD incidence from 1990 to 2021 showed an overall decrease across South Asia, with a regional AAPC of −0.061 (Table 2). Nepal had the largest decline with an AAPC of −0.372, followed by Bhutan (−0.258) and Pakistan (−0.224). Bangladesh saw a moderate reduction with an AAPC of −0.125, while India had the smallest change, with an AAPC of −0.017, indicating stable incidence rates.

**TABLE 2:** Changes in ADoD incidence and annual percent change from 1990 to 2021 in South Asian Countries.

Country	Incidence (1990)	Incidence (2021)	AAPC (with CI)
South Asia	80.57 (69.5–92.11)	79 (68.26–90.52)	−0.061^*^ (−0.070 to −0.056)
Pakistan	84.44 (73.08–97.03)	78.8 (67.99–90.38)	−0.224^*^ (−0.225 to −0.222)
Nepal	91.22 (79.31–104.24)	81.31 (70.51–93.7)	−0.372^*^ (−0.375 to −0.369)
Bhutan	83.88 (72.92–96.22)	77.45 (67.36–88.68)	−0.258^*^ (−0.261 to −0.256)
India	79.45 (68.69–90.82)	78.92 (68.29–90.58)	−0.017^*^ (−0.027 to −0.01)
Bangladesh	82.54 (71.13–94.38)	79.47 (68.72–90.87)	−0.125^*^ (−0.128 to −0.123)

^*^ Indicates that AAPC is significantly different from zero at the alpha = 0.05 level.

**FIGURE 2:**
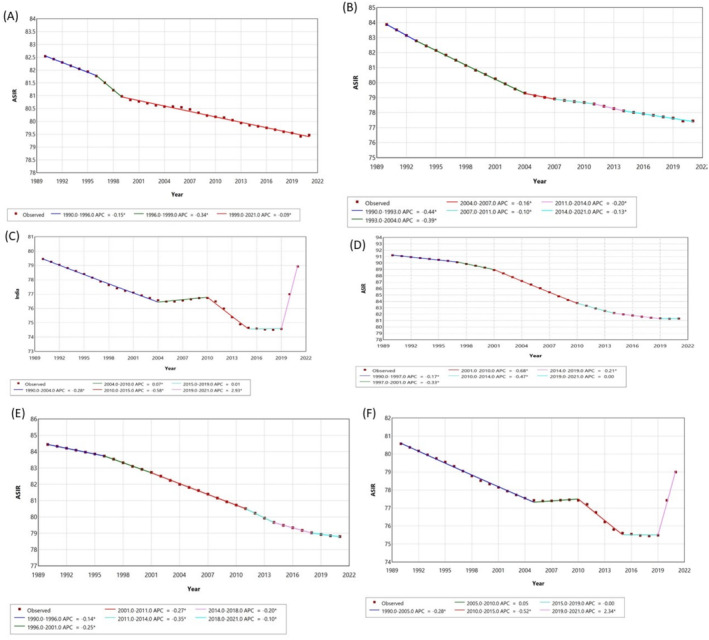
Join point regression analysis of age‐standardized incidence rates (ASIR) for Alzheimer's Disease and Other Dementias in South Asian Countries (1990–2021): (A) Bangladesh, (B) Bhutan, (C) India, (D) Nepal, (E) Pakistan, (F) South Asia.

In the South Asia Region, an initial significant decline in ASIR was observed from 1990 to 2005 with an APC of −0.2761* (95% CI: −0.2888 to −0.2649) (Figure 2). This was followed by a period of minimal change until 2010, and another sharp decline through 2015. The most notable increase was recorded in the latest segment from 2019 to 2021, where ASIR rose sharply with an APC of 2.3362* (95% CI: 2.1863–2.461). Same pattern is seen in India, particularly with a significant drop from 2010 to 2015 (APC: −0.5848*; 95% CI: −0.6582 to −0.5252) and a rapid increase from 2019 to 2021 (APC: 2.9293*; 95% CI: 2.7539–3.0793). Conversely, countries like Nepal and Bhutan exhibited consistently negative APCs across all periods, indicating a sustained decrease in ASIR, with Nepal experiencing its most significant decrease between 2001 and 2010 (APC: −0.6770*; 95% CI: −0.6851 to −0.6694).

The comparative analysis of ADoD across South Asian countries between 1990 and 2021 reveals several critical trends. The region experienced a significant 192.63% increase in ADoD cases, rising from approximately 1.76 million in 1990 to 5.15 million in 2021 (Table 3). India contributed the most substantial absolute increase, with a rise of approximately 2.82 million cases (208.84%), underscoring its dominant role in the regional trend. Bhutan recorded the highest percentage increase in cases at 218.81%, reflecting a rapid escalation in dementia prevalence. Conversely, Pakistan experienced the lowest increase in cases at 82.61%, with a relatively smaller decline in prevalence rate by 6.72%. Despite the overall rise in cases, the prevalence rates showed a consistent decline across all countries, ranging from −0.89% in India to −11.08% in Nepal.

**TABLE 3:** Comparative analysis of ADoD prevalence trend in South Asian countries (1990 vs. 2021).

Countries	No. of cases (with CI)		Percent change (with CI)	
	1990	2021	In no. of cases (1990–2021)	In rate (1990–2021)
Nepal	31930.45 (36147.61–27612.27)	83342.33 (95,053–71555.44)	161.01 (162.96–159.14)	−11.08 (−11.14 to −11.32)
Pakistan	205549.28 (235971.97–175762.76)	375360.28 (430003.15–321683.67)	82.61 (82.23–83.02)	−6.72 (−6.65 to −5.89)
Bhutan	729.14 (829.48–624.2)	2324.57 (2666.14–1996.25)	218.81 (221.42–219.81)	−8.15 (−8.55 to −7.7)
Bangladesh	170705.18 (194561.78–147048.46)	516766.09 (592095.31–446674.99)	202.72 (204.32–203.76)	−3.81 (−3.44 to −3.76)
India	1350127.81 (1543952.77–1162808.62)	4169684.39 (4772663.15–3591611.95)	208.84 (209.12–208.87)	−0.89 (−1.59 to −0.55)
South Asia	1759041.86 (2008716.24–1517058.54)	5147477.66 (5886074.83–4444260.18)	192.63 (193.03–192.95)	−2.1 (−1.82 to −2.44)

**FIGURE 3:**
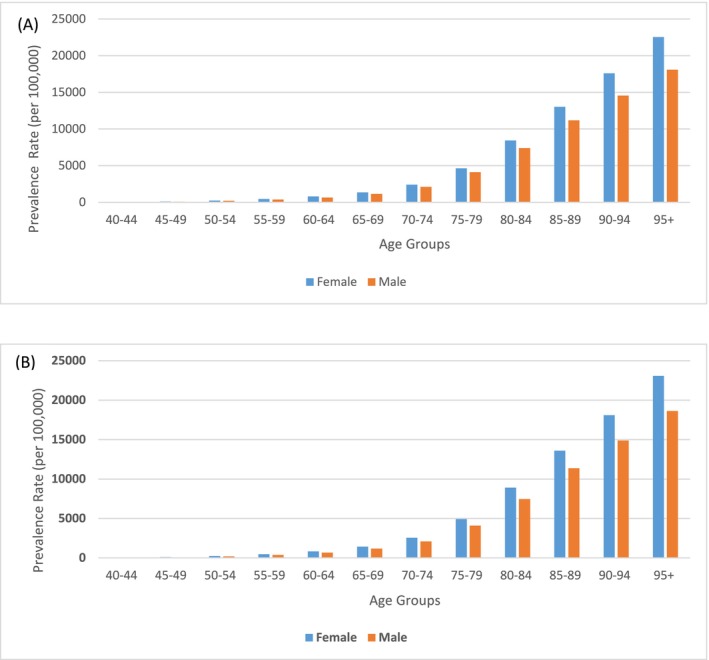
Prevalence rate of dementia by age group and gender in South Asia (A) 1990 and (B) 2021.

A comparative analysis of the prevalence rates of dementia by age group and gender in South Asia for the years 1990 and 2021(Figure 3). There was a significant increase in dementia prevalence among both genders across all age groups, with the most pronounced changes observed in the older populations. In 1990, the prevalence rates for females ranged from 15.06 per 100,000 in the 40–44 age group to 22,531.85 in the 95+ age group. By 2021, these figures had escalated to 15.23 and 23,089.27, respectively. Males showed a similar trend, starting at 13.14 per 100,000 in the 40–44 age group in 1990 and increasing to 18,083.57 in the 95+ age group, with the 2021 data showing further increases to 13.23 and 18,614.55, respectively.

**FIGURE 4:**
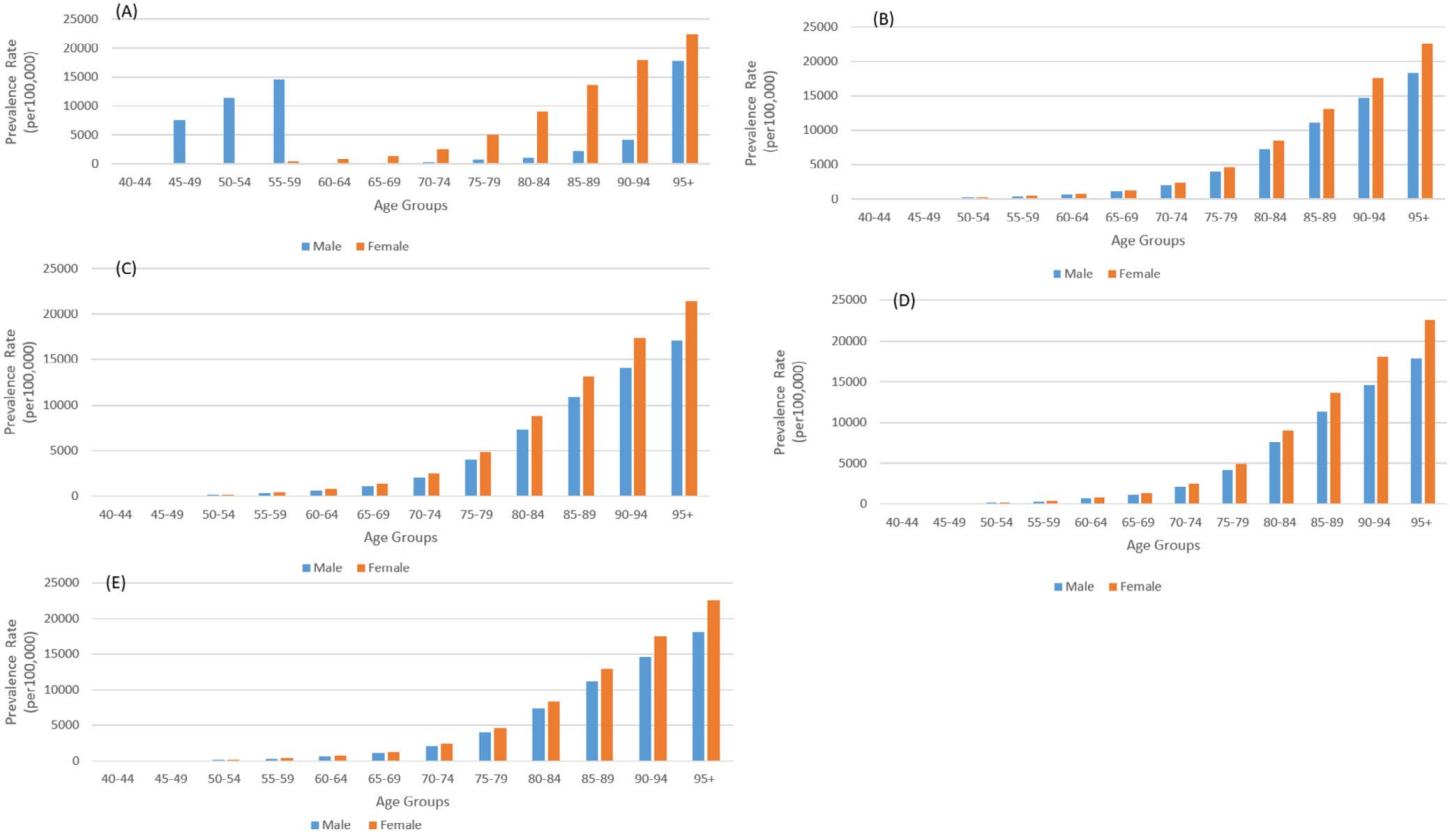
Prevalence rate of dementia by age group and gender in South Asian Countries, 2021 (A) Nepal, (B) Pakistan, (C) Bhutan, (D) Bangladesh, (E) India.

In Nepal, males start with a prevalence of 12.18 per 100,000 in the 40–44 age group, rising sharply to 17,849.30 per 100,000 in the 95+ age group, while females increase from 13.98 to 22,517.26 per 100,000 (Figure 4). This pattern of higher female prevalence is consistent across all countries. In Pakistan, males begin at 13.59 and rise to 18,359 per 100,000, while females increase from 15.10 to 22,613.50 per 100,000. Bhutan shows a lower starting prevalence of 11.85 for males and 13.68 for females, escalating to 17,048.41 and 21,373.73, respectively. Bangladesh follows a similar trend, with males starting at 11.81 and females at 13.38, increasing to 17,890.29 and 22,557.12. In India, males start at 13.23 and females at 15.28, rising to 18,073.97 and 22,525.51 in the oldest age group.

**FIGURE 5:**
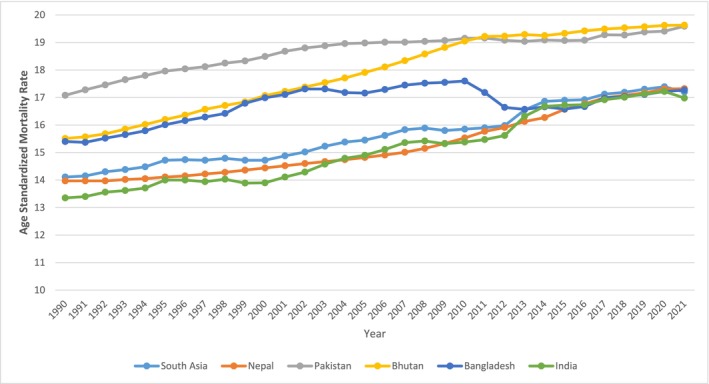
Age standardized mortality rate trends across South Asian Countries and region from 1990 to 2021.

The age‐standardized mortality rates (ASMR) in South Asia from 1990 to 2021 displayed significant regional differences and trends (Figure 5). The overall ASMR for the region rose to a peak of 18 per 100,000 in 2009 before declining to 15 per 100,000 by 2021. India and Bangladesh showed notable improvements, with India's ASMR decreasing from a peak of 17 in 2011 to 14 by 2021, and Bangladesh's dropping from 16 in 2006 to 12 by 2021. In contrast, Pakistan and Nepal experienced consistent increases in their mortality rates, with Pakistan's ASMR rising from 13 to 17 per 100,000 over the study period. Bhutan's mortality rate rose gradually but stabilized after 2018.

**FIGURE 6:**
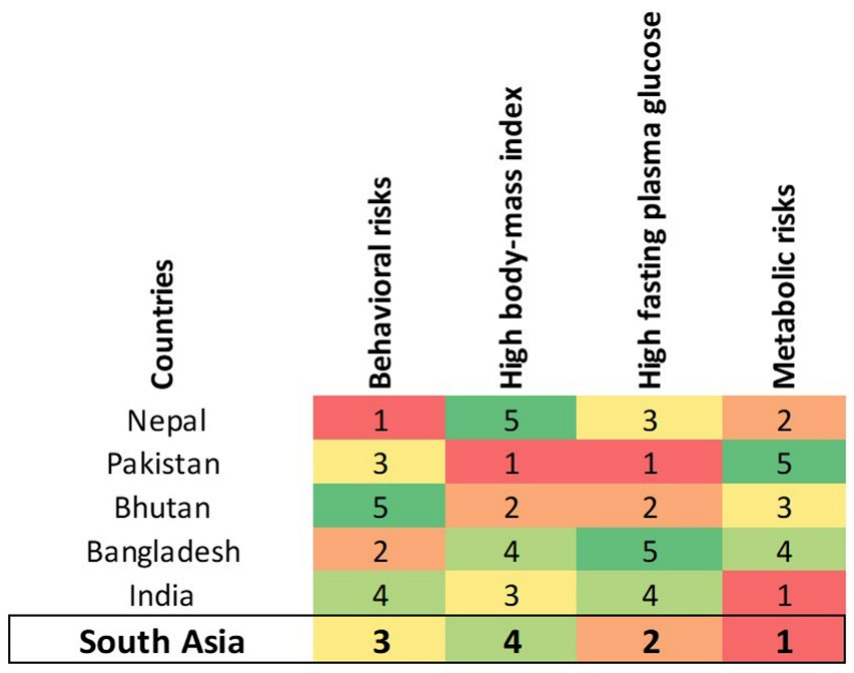
Heat map for ranking of health risk by South Asia countries for different types of risk factor of dementia.

The rankings across risk factors such as behavioral risks, high body mass index (BMI), high fasting plasma glucose (FPG), metabolic risks reveal significant regional variations (Figure 6). In South Asia, the highest‐ranking risk factor is metabolic risks, followed by high FPG. India ranks the highest for metabolic risks, while Nepal is most affected by behavioral risks. Pakistan and Bhutan is primarily impacted by high body‐mass index and high FPG. Bangladesh is most impacted by behavioral risks.

**FIGURE 7:**
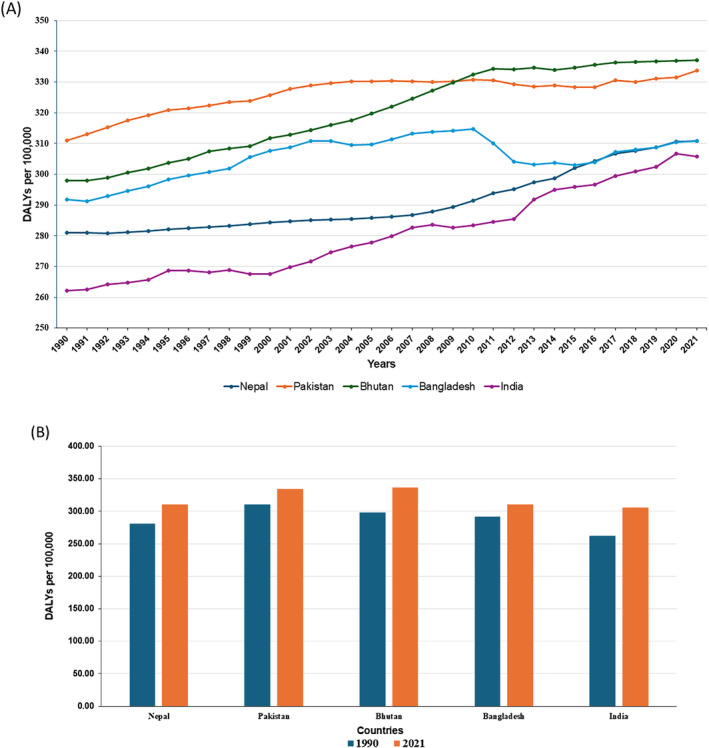
Longitudinal Analysis and Country‐Specific Comparisons of DALY Trends for Alzheimer's disease and Other Dementias in South Asia (1990–2021). (A) Longitudinal Analysis of DALY Trends for Alzheimer's disease and Other Dementias in South Asia. (B) Country‐specific DALY comparisons in South Asia: a temporal analysis.

From 1990 to 2021, DALYs due to ADoD significantly increased across five South Asian countries (Figure 7A). India experienced the most substantial rise, with DALYs increasing by 43.66 or 16.66%. Bhutan's DALYs increased by 13.14%, showing significant growth Nepal and Pakistan also experienced increases of 10.66% and 7.38%, respectively, while Bangladesh recorded the smallest rise at 6.51%. Year‐on‐year analysis revealed that DALYs in Nepal and Pakistan exhibited fluctuations with some years showing dramatic increases, in contrast to the more consistent annual increments observed in Bhutan, Bangladesh, and India, which rose annually by 0.28%, 0.14%, and 0.34%, respectively. There is a clear upward trend in DALY rates with varying rates of increase (Figure 7B). Nepal's DALY rates rose by approximately 10.66% over the 31‐year period, showcasing a consistent upward trajectory. Pakistan's DALY rates increased by about 7.38%, with intermittent fluctuations but a generally ascending pattern. Bhutan observed the most substantial growth, with a 13.14% increase, highlighted by a pronounced acceleration after 2010. Bangladesh's rates increased by approximately 6.51%, despite a mid‐period decline, ultimately concluding with a notable upward trend. India saw the highest percentage increase of 16.66%, although it experienced a slight reduction in the final year from its 2020 peak.

**FIGURE 8:**
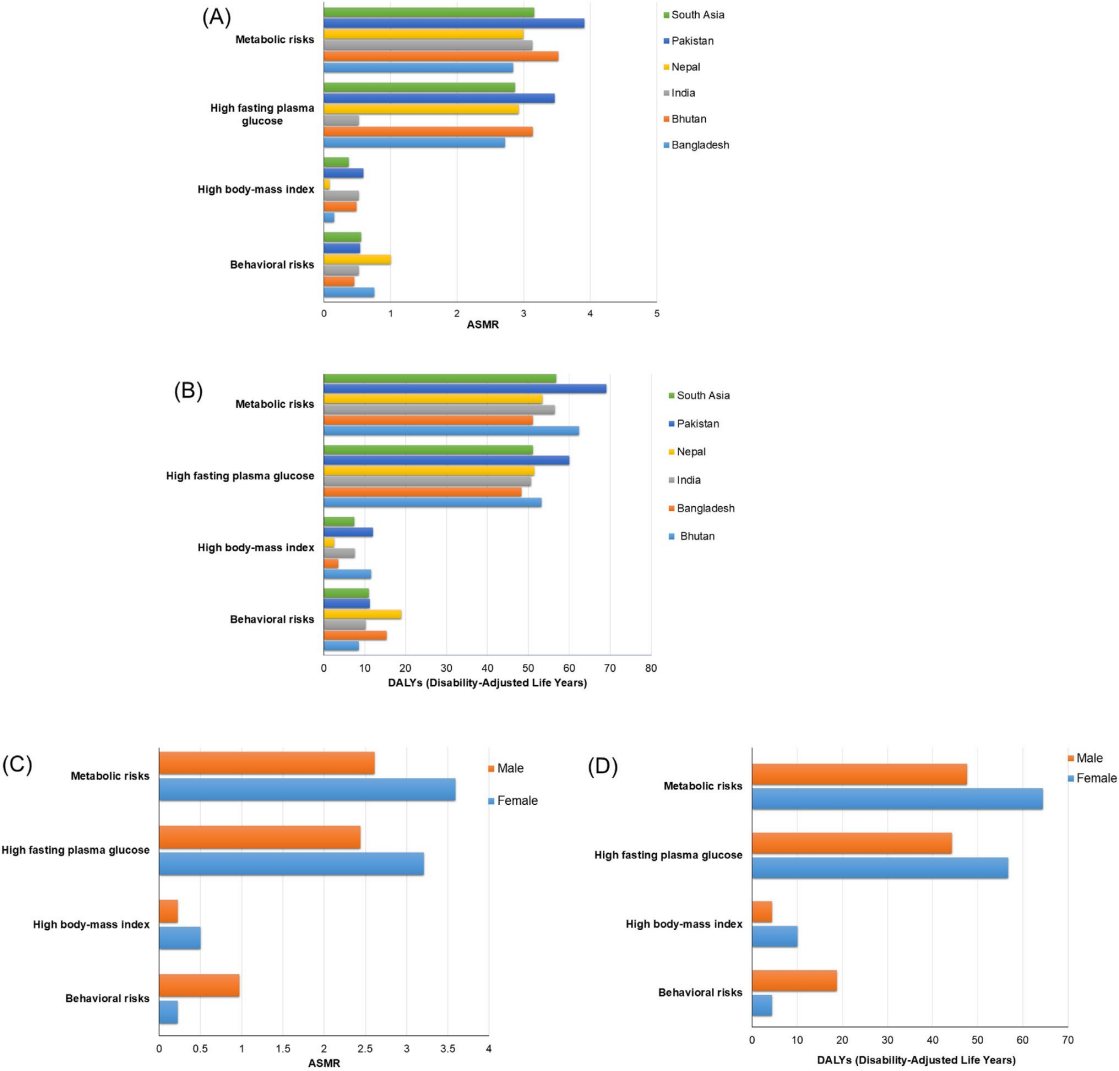
Impact of risk factors on the disease burden of Alzheimer's disease and other dementia in South Asian countries by gender, 2021. (A) Age‐standardized mortality rates by risk factor in South Asian countries. (B) DALY (Disability‐Adjusted Life Years) by risk factor in South Asian countries. (C) Age‐standardized mortality rates for key risk factors by gender. (D) DALY rates for key risk factors by gender.

Contribution of risk factors shows the significant variability by region and risk category. Behavioral risks uniformly affect the region, with Nepal registering the highest impact at an ASMR of 1.00 (Figure 8A). In contrast, metabolic risks are more severe, with Pakistan facing the greatest burden at an ASMR of 3.91. High FPG levels also vary widely, with the highest rates observed in Pakistan at 3.47, suggesting unique dietary or genetic predispositions.

DALYs associated with various risk factors demonstrates pronounced regional differences (Figure 8B). Behavioral risks and smoking‐related factors show a significant burden in Nepal, with DALYs reaching 18.86, indicating a high impact from lifestyle related behaviors. In contrast, the role of high body‐mass index varies dramatically, with Pakistan experiencing the highest DALYs at 12.01. High FPG is a major concern across the region, especially in Pakistan where it reaches 60.01 DALYs, highlighting critical challenges in managing blood glucose levels. Metabolic risks also present a severe impact, particularly in Pakistan with the highest recorded DALYs at 69.08.

Risk factors contributing to mortality in South Asia had a greater impact on females from metabolic risks, with an ASMR of 3.59 compared to 2.61 in males (Figure 8C). Specifically, females had a higher ASMR for high FPG (3.21) and high body‐mass index (0.50), suggesting a greater vulnerability to these metabolic dysfunctions than their male counterparts, who registered ASMRs of 2.44 and 0.22 for the same risk factors, respectively. Conversely, behavioral risks such as smoking and tobacco use demonstrated a stronger impact on male mortality, both presenting an ASMR of 0.97, in contrast to a much lower ASMR of 0.22 observed in females for each risk.

Males demonstrated significantly higher DALYs for behavioral risks, smoking, and tobacco use, each at 18.76, compared to females who reported considerably lower figures of 4.36 for these risk factors (Figure 8D). Conversely, females showed higher DALYs for high FPG and metabolic risks at 56.71 and 64.41, respectively, while males recorded 44.30 and 47.61 for the same factors. Notably, the impact of a high body‐mass index also differed markedly, with females experiencing 10.00 DALYs and males only 4.35.

## 4 Discussion

Dementia prevalence, incidence, mortality, and DALYs have continuously increased over the past three decades, with further increases projected [11, 20]. Currently, dementia affects approximately 0.7% of the global population, impacting 51.6 million people, a figure that has more than doubled since 1990 [18]. South Asia's aging population is driving an increase in dementia, creating a growing public health concern [20]. Dementia is an age‐related disease, and its incidence has increased significantly worldwide with increasing life expectancy [21]. Our findings align with this observation which shows the consistent pattern of increasing dementia rates with age in all countries studied. This rise is more pronounced in the elderly population, especially beyond the age of 65, where the prevalence rates surge dramatically, illustrating the significant correlation between aging and increased dementia risk. However, dementia incidence rates show varying trends across South Asia. Dementia incidence varies across South Asia, with Nepal achieving a 9.91% decline, while India's rates remain relatively stable with a marginal decrease of −0.53. The join point regression analysis further delineates periods of decline in ASIR, followed by recent increases, especially from 2019 to 2021. In contrast to the significant progress in reducing dementia incidence shown in our findings Thapa et al. highlight ongoing challenges in dementia care, including weak healthcare infrastructure, limited access to services, a lack of trained healthcare professionals, insufficient public awareness, and financial barriers for both patients and caregivers [22]. Despite these hurdles, the observed progress compared to Pakistan and India may reflect the impact of general improvements in healthcare delivery.

This recent surge in ASIR emphasizes the need for renewed focus on dementia monitoring and prevention strategies in the region. The escalating global burden of dementia, particularly AD, presents a significant challenge, with forecasts indicating an ongoing increase attributed to an aging demographic. This trend parallels the economic and societal ramifications of AD, which are comparable to those of other critical health conditions such as cancer, cardiovascular diseases, and stroke [23]. The global cost of dementia was $1.3 trillion in 2019, projected to reach $2 trillion by 2030, with informal caregiving comprising 50% of the total expenditures [14, 24]. These costs encompass direct medical expenses, social sector costs, and informal caregiving, which accounts for about 50% of total expenditures. The reliance on informal caregiving underscores the societal burden, particularly as the disease progresses, shifting costs from indirect to direct expenses and increasing the strain on families and healthcare systems, especially in LMIC [4, 14].

Other studies shows that the dementia metrics showed a continuous increase in prevalence, incidence, mortality, and DALYs rates worldwide during the last three decades, and will more than double in mortality burden over the next 20 years [25]. Women account for two‐thirds of AD cases, a statistic influenced by factors extending beyond increased life expectancy [26]. They show a higher susceptibility to ADoD due to a combination of biological, genetic, and socio cultural factors. Key factors include longer life expectancy, greater cognitive vulnerability in screening tests, faster rates of brain atrophy, potent genetic risks linked to the APOE4 allele, and hormonal changes such as estrogen decline during menopause. Additionally, chromosomal variations and the need for gender‐specific research underscore the complexity of this susceptibility [26, 27]. Targeted interventions should focus on the most affected demographics, such as the elderly and females, to mitigate the impact of dementia. In 2019, ADoD were among the top neurological disorders contributing to DALYs in Asia, with 13.5 million DALYs attributed to these conditions [28]. In South Asia, the management of dementia reveals a complex scenario where improvements in morbidity contrast sharply with rising mortality rates. Advancements in healthcare have slightly improved the YLD, indicating better management of non‐fatal impacts of dementia. However, an increase in YLL highlights the persistent challenges in addressing the terminal stages of the disease effectively. The rise in DALYs underscores the growing dementia burden, emphasizing the need for preventive and curative healthcare interventions. The variation in dementia metrics across countries like Nepal, India, and Bhutan illustrates the impact of local healthcare practices and policies on disease outcomes, with Nepal's consistent decline in incidence rates underscoring the benefits of targeted health interventions and enhanced public health infrastructure. Globally, the age‐standardized DALY rates for ADoD have shown an upward trend globally, with a 3.5% increase from 1990 to 2019 [29].

These findings underscore the necessity of holistic approach to healthcare that includes advanced diagnostics, enhanced treatment options, and improved palliative care, tailored to the cultural and socio‐economic contexts of each country to ensure effective and sustainable health interventions. There has been no effective therapy to reverse dementia thus far, and drug treatment has substantial limitations [30]. In analyzing the impact of risk factors on ADoD across South Asia, the data reveals significant regional and gender‐specific variations in ASMR and DALYs. Our finding is consistent with the other study results which shows that high FPG and metabolic risks are the most impactful across the region, particularly in Pakistan and Bhutan, highlighting a critical need for targeted interventions in dietary habits and healthcare access [31].

Smoking and tobacco use are major concerns in Nepal and Bangladesh, requiring region‐specific anti‐smoking campaigns. In Nepal, these behavioral risks significantly contribute to the disease burden, with DALYs reaching 18.86. While metabolic risks are present, their impact is less pronounced compared to regions like Pakistan. Our study focused on modifiable risk factors for dementia, such as tobacco use, smoking, metabolic risks, elevated fasting plasma glucose, and high body mass index. These factors align with global dementia prevention efforts, which highlight nine key modifiable risks: lower levels of education, hypertension, hearing loss, obesity, depression, smoking, physical inactivity, diabetes, and low social engagement. Research has also identified excessive alcohol consumption, traumatic brain injury, and air pollution as contributors to dementia [32]. Our findings emphasize the importance of lifestyle and metabolic factors in maintaining cognitive health, enhancing the broader understanding of dementia prevention. Global studies suggest that nearly 40% of dementia cases could be prevented by managing modifiable risk factors, including hypertension, smoking, low education, physical inactivity, and obesity [33].

Our findings highlight women have higher DALYs from metabolic risks (64.41 vs. 47.61 for men), indicating greater vulnerability to metabolic dysfunction. Men, on the other hand, show higher DALYs from behavioral risks. Additionally, women consistently exhibit higher dementia prevalence and ASMR, particularly in older age groups and due to metabolic factors, further emphasizing their higher burden of dementia. Other global studies also support these findings. Research by Kim et al. and Tanaka et al. highlights that women are more vulnerable to metabolic risks, such as diabetes, which significantly increases dementia risk [34, 35]. While our findings underscore the greater metabolic burden in women, factors like lifestyle, genetics, and healthcare access also contribute to these disparities. This suggests that public health strategies should prioritize metabolic health for women and smoking cessation for men, while addressing broader risk factors to improve dementia outcomes across genders. Targeted interventions should focus on the most affected demographics, such as the elderly and females, to mitigate the impact of dementia.

Our study encounters two main limitations. First, the reliance on generalized models and assumptions may not capture the unique and detailed epidemiological features of dementia specific to the region, potentially leading to inaccurate estimates of the disease burden. Second, the consistency and accuracy of dementia data, especially from low and middle‐income countries, present challenges due to variations in diagnostic criteria and data collection standards, which can impact the reliability of our findings.

## 5 Conclusion

This study on ADoD in South Asia from 1990 to 2021 provides significant insights into the regional trends and challenges with only a decrease in incidence rates from 80.57 to 79 per 100,000 and a concerning rise in mortality rates from 14.11 to 17.2 per 100,000. Disability‐Adjusted Life Years also increased from 272.02 to 308.27 per 100,000, highlighting an escalating burden. This study emphasizes the importance of implementing gender‐specific health interventions in South Asia, with a focus on addressing metabolic risks more aggressively in females and behavioral risks in males to reduce mortality more effectively. Furthermore, the significant contribution of metabolic risks to the growing dementia burden, particularly in Pakistan and Bangladesh, highlights the pressing need for public health interventions that are tailored to the regional health profiles and risk factors, ensuring more targeted and effective outcomes.

## Author Contributions

Conceptualization: Shubham Chauhan, Diptismita Jena; data curation: Shubham Chauhan, Shilpa Gaidhane. Formal analysis: Navneet Devand, Ganesh Bushi. Investigation: G. Padma Priyaand, Pawan Sharma. Methodology: Mahakshit Bhat. Visualization: Sanjit Sah. Supervision: M. Ravi Kumar and Aashna Sinha. Writing – original draft: Quazi Syed Zahiruddin, Muhammed Shabil, Shubham Chauhan. Writing – review and editing: Shubham Chauhan, Diptismita Jena, Shilpa Gaidhane, Navneet Devand, Ganesh Bushi, G. Padma Priyaand, Pawan Sharma, Mahakshit Bhat, Sanjit Sah, M. Ravi Kumar, Aashna Sinha, Quazi Syed Zahiruddin, Muhammed Shabil, (SSAH), Rukshar Syed, Kamal Kundra, Alisha Dash, Hashem Abu Serhan. All authors revised and approved the final manuscript.

## Ethics Statement

Ethical approval was not required for this study as the data used were publicly available and de‐identified from the GBD database.

## Consent

The authors have nothing to report.

## Conflicts of Interest

The authors declare no conflicts of interest.

## Data Availability

The data that support the findings of this study are openly available in https://vizhub.healthdata.org/gbd‐results/ at reference number GBD 2021.

## References

[1] E. S. Oh and P. V. Rabins , “Dementia,” Annals of Internal Medicine 171, no. 5 (2019): Itc33, 10.7326/aitc201909030. 31476229

[2] A. Nandi , N. Counts , S. Chen , et al., “Global and Regional Projections of the Economic Burden of Alzheimer's Disease and Related Dementias From 2019 to 2050: A Value of Statistical Life Approach,” eClinical Medicine 51, no. 51 (2022): 101580, 10.1016/j.eclinm.2022.101580. PMC9310134 35898316

[3] X. Li , X. Feng , X. Sun , N. Hou , F. Han , and Y. Liu , “Global, Regional, and National Burden of Alzheimer's Disease and Other Dementias, 1990–2019,” Frontiers in Aging Neuroscience 14 (2022): 937486, 10.3389/fnagi.2022.937486. 36299608 PMC9588915

[4] Health TLP , “Will Dementia Hamper Healthy Ageing?,” Lancet Public Health 7, no. 2 (2022): e93, 10.1016/s2468-2667(22)00009-3. 35074138

[5] C. G. Lyketsos , M. C. Carrillo , J. M. Ryan , et al., “Neuropsychiatric Symptoms in Alzheimer's Disease,” Alzheimer's & Dementia 7, no. 5 (2011): 532–539, 10.1016/j.jalz.2011.05.2410. PMC3299979 21889116

[6] A. Pless , D. Ware , S. Saggu , H. Rehman , J. Morgan , and Q. Wang , “Understanding Neuropsychiatric Symptoms in Alzheimer's Disease: Challenges and Advances in Diagnosis and Treatment,” Frontiers in Neuroscience 17 (2023): 1263771, 10.3389/fnins.2023.1263771. 37732300 PMC10508352

[7] S. Srivastava , R. Ahmad , and S. K. Khare , “Alzheimer's Disease and Its Treatment by Different Approaches: A Review,” European Journal of Medicinal Chemistry 216 (2021): 113320, 10.1016/j.ejmech.2021.113320. 33652356

[8] M. Amini , F. Zayeri , and S. S. Moghaddam , “Years Lived With Disability due to Alzheimer's Disease and Other Dementias in Asian and North African Countries: A Trend Analysis,” Journal of Epidemiology and Global Health 9, no. 1 (2019): 29, 10.2991/jegh.k.190305.002. 30932387 PMC7310755

[9] T. Ahmed , K. Kumar , and P. Zhang , “A Systematic Review of the Status of Neuropsychological Research and Dementia in South Asia,” Discover Psychology 3, no. 1 (2023): 16, 10.1007/s44202-023-00078-2.

[10] T. F. Ahmed and A. Ahmed , “The Road to Crisis: State of Pakistan's Research Output in Dementia,” Journal of the Pakistan Medical Association 73, no. 11 (2023): 2298, 10.47391/JPMA.9548. 38013555

[11] E. Nichols , J. D. Steinmetz , S. E. Vollset , et al., “Estimation of the Global Prevalence of Dementia in 2019 and Forecasted Prevalence in 2050: An Analysis for the Global Burden of Disease Study 2019,” Lancet Public Health 7, no. 2 (2022): e105–e125, 10.1016/s2468-2667(21)00249-8. 34998485 PMC8810394

[12] N. Farina , J. Rajagopalan , S. Alladi , et al., “Estimating the Number of People Living With Dementia at Different Stages of the Condition in India: A Delphi Process,” Dementia (London, England) 23, no. 3 (2024): 438–451, 10.1177/14713012231181627. 37272749 PMC11041066

[13] N. Farina , R. Jacobs , Y. Turana , et al., “Comprehensive Measurement of the Prevalence of Dementia in Low‐ and Middle‐Income Countries: STRiDE Methodology and Its Application in Indonesia and South Africa,” British Journal of Psychiatry Open 9, no. 4 (2023): e102, 10.1192/bjo.2023.76. 37278200 PMC10305093

[14] A. Wimo , K. Seeher , R. Cataldi , et al., “The Worldwide Costs of Dementia in 2019,” Alzheimer's & Dementia 19, no. 7 (2023): 2865–2873, 10.1016/S2468-2667(21)00249-8. PMC10842637 36617519

[15] E. Nichols and T. Vos , “The Estimation of the Global Prevalence of Dementia From 1990 to 2019 and Forecasted Prevalence Through 2050: An Analysis for the Global Burden of Disease (GBD) Study 2019,” Alzheimer's & Dementia 17 (2021): 12–14, 10.1016/S2468-2667(21)00249-8.

[16] C. J. L. Murray , “The Global Burden of Disease Study at 30 Years,” Nature Medicine 28, no. 10 (2022): 2019–2026, 10.1038/s41591-022-01990-1. 36216939

[17] E. Nichols , C. Szoeke , S. E. Vollset , et al., “Global, Regional, and National Burden of Alzheimer's Disease and Other Dementias, 1990–2016: A Systematic Analysis for the Global Burden of Disease Study 2016,” Lancet Neurology 18, no. 1 (2019): 88–106, 10.1016/S1474-4422(18)30403-4. 30497964 PMC6291454

[18] R. T. Vieira , L. Caixeta , S. Machado , et al., “Epidemiology of Early‐Onset Dementia: A Review of the Literature,” Clinical Practice and Epidemiology in Mental Health: CP & EMH 9 (2013): 88–95, 10.2174/1745017901309010088. 23878613 PMC3715758

[19] G. A. Jicha and S. A. Carr , “Conceptual Evolution in Alzheimer's Disease: Implications for Understanding the Clinical Phenotype of Progressive Neurodegenerative Disease,” Journal of Alzheimer's Disease 19, no. 1 (2010): 253–272, 10.3233/jad-2010-1237. PMC2881218 20061643

[20] N. Mukadam , F. J. Wolters , S. Walsh , et al., “Changes in Prevalence and Incidence of Dementia and Risk Factors for Dementia: An Analysis From Cohort Studies,” Lancet Public Health 9, no. 7 (2024): e443–e460, 10.1016/S2468-2667(24)00120-8. 38942556

[21] R. Power , A. Prado‐Cabrero , R. Mulcahy , A. Howard , and J. M. Nolan , “The Role of Nutrition for the Aging Population: Implications for Cognition and Alzheimer's Disease,” Annual Review of Food Science and Technology 10 (2019): 619, 10.1146/annurev-food-030216-030125. 30908950

[22] P. Thapa , K. Marahatta , S. Upadhyay Raj , et al., “Dementia Care Landscape in Nepal: Understanding the Context, Barriers, and Opportunities for the Development of a National Dementia Care Plan,” International Journal of Geriatric Psychiatry 39, no. 6 (2024): e6111, 10.1002/gps.6111. 38862409

[23] WHO , “World Alzheimer Report 2019,” (2019).

[24] L. X. Tay , S. C. Ong , L. J. Tay , T. Ng , and T. Parumasivam , “Economic Burden of Alzheimer's Disease: A Systematic Review,” Issues 40 (2024): 1, 10.1016/j.vhri.2023.09.008. 37972428

[25] S. Javaid , C. Giebel , M. Khan , and M. Hashim , “Epidemiology of Alzheimer's Disease and Other Dementias: Rising Global Burden and Forecasted Trends,” F1000Res 10 (2021): 425, 10.12688/f1000research.50786.1.

[26] J. Martinkova , F.‐C. Quevenco , H. Karcher , et al., “Proportion of Women and Reporting of Outcomes by Sex in Clinical Trials for Alzheimer Disease: A Systematic Review and Meta‐Analysis,” JAMA Network Open 4, no. 9 (2021): e2124124, 10.1001/jamanetworkopen.2021.24124. 34515784 PMC12243614

[27] K. A. Lin and P. M. Doraiswamy , “When Mars Versus Venus Is Not a Cliché: Gender Differences in the Neurobiology of Alzheimer's Disease,” Frontiers in Neurology 5 (2015): 288, 10.3389/fneur.2014.00288. 25628598 PMC4290582

[28] Y. Wang , J. Liang , Y. Fang , et al., “Burden of Common Neurologic Diseases in Asian Countries, 1990–2019,” Neurology 100, no. 21 (2023): e2141, 10.1212/WNL.0000000000207218. 37015823 PMC10238164

[29] C. Ding , Y. Wu , X. Chen , et al., “Global, Regional, and National Burden and Attributable Risk Factors of Neurological Disorders: The Global Burden of Disease Study 1990–2019,” Frontiers in Public Health 10 (2022): 952161, 10.3389/fpubh.2022.952161. 36523572 PMC9745318

[30] L. N. Gitlin , H. C. Kales , and C. G. Lyketsos , “Nonpharmacologic Management of Behavioral Symptoms in Dementia,” JAMA: The Journal of the American Medical Association 308, no. 19 (2012): 2020–2029, 10.1001/jama.2012.36918. 23168825 PMC3711645

[31] D. L. Palliyaguru , N. Palliyaguru , C. V. Ligo Teixeira , et al., “Status, Determinants and Risk Factors of All‐Cause Dementia in South Asia: Findings From a Preliminary Analysis of Global Health Data,” preprint, medRxiv, March 7, 2024, 10.1101/2024.03.06.24303854.

[32] G. Livingston , J. Huntley , A. Sommerlad , et al., “Dementia Prevention, Intervention, and Care: 2020 Report of the Lancet Commission,” Lancet 396, no. 10248 (2020): 413–446, 10.1016/S0140-6736(20)30367-6. 32738937 PMC7392084

[33] L. M. Nakakogue and R. L. Fonseca , “Prevalence of Risk Factors for Dementia and Association With Cognitive Alterations in an Elderly Population From the South of Brazil,” Alzheimer's & Dementia 19 (2023): e073048, 10.1002/alz.073048.

[34] Y. H. Kim , N. H. Kim , M. H. Jung , and H. J. Kim , “Sex Differences in Metabolic Risk Indicator of Dementia in an Elderly Urban Korean Population: A Community‐Based Cross‐Sectional Study,” Geriatrics & Gerontology International 17, no. 11 (2017): 2136–2142, 10.1111/ggi.13049. 28509397

[35] M. Tanaka , H. Imano , M. Hayama‐Terada , et al., “Sex‐ and Age‐Specific Impacts of Smoking, Overweight/Obesity, Hypertension, and Diabetes Mellitus in the Development of Disabling Dementia in a Japanese Population,” Environmental Health and Preventive Medicine 28 (2023): 11, 10.1265/ehpm.22-00187. 36740267 PMC9922560

